# Characterization of *Salmonella* phage of the genus *Kayfunavirus* isolated from sewage infecting clinical strains of *Salmonella enterica*

**DOI:** 10.3389/fmicb.2024.1391777

**Published:** 2024-06-03

**Authors:** Ramya Juliet, Archana Loganathan, Ayyanraj Neeravi, Yamuna Devi Bakthavatchalam, Balaji Veeraraghavan, Prasanth Manohar, Ramesh Nachimuthu

**Affiliations:** ^1^School of Biosciences and Technology, Vellore Institute of Technology, Vellore, India; ^2^Department of Clinical Microbiology, Christian Medical College, Vellore, India; ^3^Department of Biochemistry and Biophysics, Texas A&M AgriLife Research, Texas A&M University, College Station, TX, United States; ^4^Center for Phage Technology, Texas A&M AgriLife Research, Texas A&M University, College Station, TX, United States

**Keywords:** *Salmonella*, food biocontrol, drug resistance, bacteriophages, phage therapy

## Abstract

The emergence of multi-drug resistance in *Salmonella*, causing food-borne infections, is a significant issue. With over 2,600 serovars in in *Salmonella* sp., it is crucial to identify specific solutions for each serovar. Phage therapy serves as an alternate treatment option. In this study, vB_SalP_792 phage was obtained from sewage, forming plaques in eight out of 13 tested clinical *S. enterica* isolates. Transmission electron microscopy (TEM) examination revealed a T7-like morphotype. The phage was characterized by its stability, life cycle, antibiofilm, and lytic ability in food sources. The phage remains stable throughout a range of temperatures (−20 to 70°C), pH levels (3–11), and in chloroform and ether. It also exhibited lytic activity within a range of MOIs from 0.0001 to 100. The life cycle revealed that 95% of the phages attached to their host within 3 min, followed by a 5-min latent period, resulting in a 50 PFU/cell burst size. The vB_SalP_792 phage genome has a dsDNA with a length of 37,281 bp and a GC content of 51%. There are 42 coding sequences (CDS), with 24 having putative functions and no resistance or virulence-related genes. The vB_SalP_792 phage significantly reduced the bacterial load in the established biofilms and also in egg whites. Thus, vB_SalP_792 phage can serve as an effective biocontrol agent for preventing *Salmonella* infections in food, and its potent lytic activity against the clinical isolates of *S. enterica*, sets out vB_SalP_792 phage as a successful candidate for future *in vivo* studies and therapeutical application against drug-resistant Salmonella infections.

## Introduction

*Salmonella enterica* is a facultative intracellular Gram-negative pathogen that belongs to the *Enterobacteriaceae* family. It has six sub-species and more than 2,500 serovars (Popoff et al., 2004; Grimont and Weill, 2007). Consuming contaminated foods such as eggs, milk, poultry, and pork can lead to gastrointestinal diseases (Crump et al., 2008; Pires et al., 2014). Both *S. enterica serovar Typhimurium* and *Enteritidis* are responsible for multiple epidemics (Crump and Mintz, 2010). Salmonellosis is a rising problem in many nations, in addition to typhoid fever (Majowicz et al., 2010). *Salmonella* infections commonly present with symptoms such as diarrhea, fever, nausea, vomiting, headache, and stomach pain (Staes et al., 2019). Multi-drug resistant *Salmonella* infections are on the rise because of the inappropriate and excessive use of antibiotics, particularly in chickens and agriculture.

*Salmonella enterica* infections are commonly managed with fluoroquinolones and third-generation cephalosporins (Feasey et al., 2015; Wong et al., 2015). Upon the emergence of resistance to first-line antibiotic regimens such as chloramphenicol, amoxicillin, and co-trimoxazole in *Salmonella*; extended-spectrum cephalosporins, and fluoroquinolones were considered as a treatment option for salmonellosis (Hsu et al., 2023). Following this, ciprofloxacin resistance in *Salmonella*, which emerged as a global threat, prompted the World Health Organization to categorize *Salmonella* under the ‘High priority pathogen’ list (Asokan et al., 2019). However, the prevalence of resistance to a last-resort drug like carbapenem is rare in *Salmonella*, but the identification of carbapenemase enzyme in Non-typhoidal *Salmonella* has also been reported (Fernández et al., 2018).

By 2050, antibiotic-resistant infections are predicted to cause approximately 10 million deaths worldwide. An average of 260,000 people die every year due to food-related infections (Łojewska and Sakowicz, 2021). The incidence of gastroenteritis caused by *Salmonella* is 93 million cases leading to 155,000 deaths globally (Castro-Vargas et al., 2020). The Center for Disease Control and Prevention reports 420 deaths, 26,500 hospitalizations, and 1.35 million cases of Salmonellosis in the United States every year because of the consumption of contaminated food (Scallan et al., 2011; Gruenberg, 2023; CDC, 2024). Additionally, several studies report the emergence of multidrug resistance in *Salmonella* infections (Singh et al., 2010; Akullian et al., 2018; Dieye et al., 2022) and outbreaks of extensively drug-resistant *Salmonella* infections drive the scientific community to search for new treatment methods (Medalla et al., 2017; Wei et al., 2019). The shortage in the discovery of new antibiotics in the pipeline, and the ceaseless unfolding of multiple drug-resistant mechanisms in bacteria, forces medical practitioners to supplement antibiotics with either vaccines, monoclonal antibodies, peptides, phytochemicals, antimicrobial enzymes, probiotics, plant-based products, phages or phage-derived enzymes to manage resistant infections (Łojewska and Sakowicz, 2021).

Bacteriophages or phages, are prokaryotic viruses that infect and kill bacteria. They are the most prevalent living organisms on Earth (Kutateladze and Adamia, 2010; Kim et al., 2019). Phage therapy involves using live phages to treat bacterial illnesses and has been effectively utilized for more than a century in nations such as the Soviet Union (now Russia), Georgia, and Poland (Yang et al., 2023). Phages were used to cure bacterial infections in the pre-antibiotic era, and with the discovery of antibiotics, the interest in phage therapy has diminished. The renewed interest and increased focus on phage research have enhanced our understanding of phage-bacterial interactions and filled the knowledge gap in therapeutic phage synthesis. Advancements in phage therapy have shown promise as an alternative to antibiotics, with positive outcomes observed in laboratory and animal studies, as well as in limited clinical trials (Eskenazi et al., 2022; Dedrick et al., 2023; Onallah et al., 2023). Phages offer advantages such as species-/strain-specificity, auto-dosing, non-toxicity to mammalian cells, and strong anti-biofilm properties (Kutateladze and Adamia, 2010; Kim et al., 2019). Phage therapy in clinical settings is being investigated due to a lack of understanding of pharmacodynamics and pharmacokinetics, despite the common usage of phages in the food industry nowadays (Chegini et al., 2021).

In 2017, a patient in the United States who received phage therapy for a multi-drug resistant *A. baumannii* infection fully recovered from his systemic infection (Hitchcock et al., 2023). This case study showcased phage therapy’s capability as a therapeutic application. A series of successful case studies thereafter have been reported from phage therapy trials since 2018 against various multi-drug resistant pathogens such as *Achromobacter*, *Acinetobacter baumannii*, *Klebsiella pneumoniae*, *E. coli*, *Mycobacterium abscessus*, *Enterococcus faecalis*, *and Staphylococcus aureus* (Petrovic Fabijan et al., 2023). Bacteriophages are getting attention from researchers as a viable strategy for the biological control of *Salmonella* infections because of their safety, effectiveness, and specificity (Goodridge and Bisha, 2011; Pereira et al., 2016). Due to the variety of *Salmonella* serotypes, it is crucial to isolate and analyze a wide range of phages for potential therapeutic use. This study isolated a lytic phage vB_SalP_792 from sewage water and described it based on morphology, physio-chemical stability, life cycle, and genomic analysis. Furthermore, the phage’s capability to survive in eggs was assessed.

## Materials and methods

### Ethical approval

Ethical approval was from the Institutional Ethical Committee for Studies on Human Subjects (IECH), ref. no. VIT/IECH/004/Jan28.2017.

### Bacterial strains and culture conditions

Clinical isolates of various bacteria were collected from the Hi-Tech Diagnostic Center in Chennai, Tamil Nadu, India. The bacteria included *S. enterica* (*n* = 13), *E. coli* (*n* = 10), *K. pneumoniae* (*n* = 10), *A. baumannii* (*n* = 10), *P. aeruginosa* (*n* = 10), *Serratia* (*n* = 1), *Citrobacter* (*n* = 2), *Proteus* (*n* = 7), *S. aureus* (*n* = 10), *Streptococcus* (*n* = 2), and *Enterococcus* (*n* = 1). The isolates were identified using the VITEK identification system and additional investigations were conducted at the Antibiotic Resistance and Phage Therapy Laboratory, VIT, Vellore. The isolates were sub-cultured in Luria Bertani (LB) (Hi-Media, India) medium and stored in a 10% glycerol stock at −20°C for further use.

### Isolation and propagation of phages

The host bacteria were cultured in LB media for standard phage isolation and propagation experiments. The sewage water sample from the Vellore area (12°58′12.2″ N 79°09′33.9″ E) was collected for phage isolation and processed using the phage enrichment technique with Sal_01 *Salmonella* clinical strain as host. In brief, to an exponentially growing bacterial culture, a sewage sample was added and incubated overnight at 37°C while shaking at 120 rpm. The sample was centrifuged at 6,000 × *g* for 15 min and the supernatant was filtered through a 0.22 μm syringe filter (Paal LifeSciences) and the filtrate was spotted onto a bacterial lawn of Sal_01. Next, a double agar overlay was performed to verify the presence of phages. Briefly, 200 μL of culture in logarithmic phase (OD_600_ nm of 0.1), 100 μL of phage filtrate, and 4 mL of molten soft agar (0.45%) were added and poured onto a hard agar plate. The plates were incubated for 16 h at 37°C. The presence of plaques confirmed the phage infectivity and the morphology of the plaques was observed (Prasanth et al., 2021).

### Purification of lytic phage and propagation

Following enrichment, phages were isolated and purified using a plaque assay to obtain a homogeneous population. In brief, one clear plaque was chosen from a double agar overlay plate using the pickate technique and grown on a host strain. The technique was repeated thrice to purify the phages. 4 mL of SM buffer [5.8 g NaCl, 50 mL 1 M Tris–HCl (pH 7.5), 2 g MgSO_4_.7H_2_O, and 5 mL of 2% gelatin for 1,000 mL] was added to the plate containing purified plaques. The mixture was left at room temperature for 4 h without agitation. The buffer was extracted from the plate using a pipette and then centrifuged at 6,000 × *g* for 15 min. The resulting supernatant was combined with the host bacteria for growth in broth, incubated for 24 h, and then centrifuged again to collect the supernatant. The phages were replicated and then filtered using a 0.22 μm-sized syringe filter. The titer was measured in plaque-forming units (PFU) and the phages were kept at 4°C for future use.

### Host range analysis and efficiency of plating

A spot test was initially employed to determine the host range of the phage. The phage was isolated specifically targeting the *S. enterica* isolate Sal_01, and its ability to infect other strains was tested against 13 non-repetitive *Salmonella enterica* isolates. Briefly, a phage lysate (10^8^ PFU/mL) was spotted on the bacterial lawn and incubated overnight at 37° C. The plates were then observed for spot clearance as described previously (Shang et al., 2021).

The EOP was determined using a double agar overlay method, and the PFU/mL was calculated. The EOP is the phage’s capacity to plaque or infect, with a ratio of PFU/mL against the host bacteria compared to the test bacterium. It is considered “high” if the ratio exceeds 50% and “low” if it is below 50% (Manohar et al., 2018).

### Microscopic analysis

Phage morphology was examined using transmission electron microscopy (TEM). Briefly, 10 μL of phage lysate (10^8^ PFU/mL) was applied to a copper grid and negatively stained using 1% (w/v) uranyl acetate for 1 min. The grid was then rinsed twice with sterile water (to remove the excess stain), air-dried, and observed under a transmission electron microscope (FEI-TECNAI G2-20 TWIN, VIT, Vellore) (Prasanth et al., 2021).

### Phage stability studies

The phages were tested for their survival rates under varying temperatures and pH levels. To assess thermal stability, 100 μL of the phage lysate (10^10^ PFU/mL) was exposed to different temperatures (−20, 0, 4, 20, 37, 40, 50, 60, 70, and 80°C) for 1 h. For pH stability experiments, the SM buffer was adjusted to pH levels of 3, 5, 7, 9, and 11. Phages were added at a final concentration of 10^10^ PFU/mL and incubated at 37°C for 1 h. Phage stability was tested in solvents including 100% chloroform, 100% ethanol, and 100% ether. The phages were incubated for 1 h at 37°C in each solvent. Phage lytic activity for stability studies was assessed using the double agar overlay method. Reduction in PFU/mL was calculated and a graph was plotted (Manohar et al., 2018; Prasanth et al., 2021).

### Life cycle

The adsorption assay involved titrating the quantity of free phages to establish the duration required for the phages to adhere to the bacterial cell (Manohar et al., 2018). Briefly, phage lysate at a ratio of 0.1 to the logarithmic phase bacteria (10^8^ CFU/mL) was introduced, with the phage lysate concentration of 10^7^ PFU/mL (equals MOI of 0.1). Every minute, 100 μL aliquots of the bacteriophage suspension were treated with 1% chloroform throughout a 5-min period. Subsequently, a double agar overlay technique was used for titration. All the experiments were performed three times and data analysis was done using GraphPad Prism software (Manohar et al., 2018).

One-step phage growth was used to measure the latent period and burst size. To determine the latency period (time taken post-adsorption to lysis) of the phage, 1 mL of host bacteria (10^8^ CFU/mL) was mixed with phage lysate of MOI 0.1 (10^7^ PFU/mL). The phage particles were left to attach for 3 min (pre-determined adsorption time) and then centrifuged at 10,000 × *g* for 5 min. The supernatant containing the free phages was discarded and the pellet was resuspended with 10 mL of fresh LB broth and incubated at 37°C. Aliquots were collected every 3 min intervals for a total of 15 min and titrated using the double agar overlay method. The number of phage particles released from one infected bacterial cell is known as the burst size. The burst size was calculated by subtracting the initial PFU/ml from the first burst. Each experiment was conducted three times and a graph was plotted to determine the latent period (Manohar et al., 2018).

### *In vitro* phage kinetics

The *in vitro* lytic activity of vB_SalP_792 was assessed at various MOIs ranging from 0.0001 to 100. In brief, 100 μL of bacteria (OD_600_ nm of 0.08) was mixed with an equal volume of phage lysate ranging from 10^4^ to 10^10^ PFU/mL in a 96-well microtiter plate to achieve various MOIs from 0.0001 to 100, and then incubated at 37°C. The controls used were bacteria without phages and a growth medium. Measurements were recorded (OD 600 nm) every 2 h for a total of 20 h using an ELISA microplate reader (Bio-Rad). All the experiments were conducted three times and the growth kinetics were graphed (Shang et al., 2021).

### Determining the stability and activity of phage in egg

*Salmonella’s* significance as a food-borne pathogen led to testing the survival of phages in chicken eggs. The eggshell was first disinfected by washing with distilled water and 75% ethanol, then treated with UV light for 30 min (Zhang et al., 2021). The egg white and yolk were aseptically separated and used for the study. Egg white and yolk were spotted on the LB agar plate for sterility verification. The stability of the phage was assessed in both egg white and egg yolk by incubating it with phage (10^10^ PFU/mL) for 1 h and then titrating it using the double agar overlay method. The phage’s activity in the egg was determined by incubating bacteria (10^8^ CFU/mL) and phage (10^8^ PFU/mL) in egg white and egg yolk for 1 h. Changes in CFU and PFU were quantified. The egg white and yolk incubated with the bacteria only and the phage only served as controls in the experiment.

### Determining the anti-biofilm activity of phage

The phage’s ability to degrade biofilms was tested on eight biofilm-producing *S. enterica* clinical isolates. A biofilm study was conducted using a procedure described elsewhere with some modifications (Loganathan and Nachimuthu, 2022). Briefly, a single colony of bacterial culture was inoculated into LB broth and incubated at 37° C for 24 h. The overnight culture was adjusted to 0.5 MacFarland turbidity and then diluted 1:100. Subsequently, 100 μL was added to a 96-well flat bottom microtiter plate. The plate was incubated for 24 h at 37° C. After incubation, the media containing planktonic cells was removed, and 100 μL of phage (1 × 10^9^ PFU/ mL) was added to the established biofilms and incubated for an additional 24 h. The biofilm was quantified using crystal violet assay at OD_570_ nm. The control group consisted of bacterial biofilm without phage treatment and LB broth.

### Phage DNA extraction and genome sequencing

The phage DNA was isolated using the phenol-chloroform method (Manohar et al., 2022). Briefly, the lysate was treated with 1% chloroform and then passed through a syringe filter (0.22 μ) to remove the bacteria. The purified phage lysate was treated with DNase I and RNase, and incubated at 37°C for 1 h. The phage was then treated with 0.5 M EDTA, Proteinase K, and 10% SDS for 1 h at 55°C. The DNA was precipitated using ethanol and then the pellet was resuspended in TE buffer.

The genome was sequenced using the Illumina NovaSeq 6000 platform at Unipath Specialty Laboratory, Ahmedabad, Gujarat, India. FastQC (version 0.11.9) (Andrews, 2010) was used to assess the quality of the reads. FastP (version 0.20.1) (Chen et al., 2018) was utilized to remove adaptors from the raw reads. The raw reads were *de novo* assembled using SPAdes (v3.15) (Bankevich et al., 2012) according to the Shovill pipeline 1.1.0. The assembled genome was quality-checked using CheckV (v1.0.1) (Nayfach et al., 2021). Open Reading Frames (ORFs) were predicted using Prodigal v2.11.0-gv (Hyatt et al., 2010). The identified ORFs were annotated using NCBI Blastp with an *e*-value threshold of 10^−5^. PhageTerm (phagetermvirome-4.3) (Garneau et al., 2017) was used to determine the packaging type. PhaBox web server was used for predicting the phage lifecycle (Shang et al., 2023). Resistance genes and mobile genetic elements were identified using AMRFinder (AMRFinderPlus v3.11.2026), Resfinder 4.1, and Mobile Element Finder 1.0.3 CLI tools. The TMHMM server 2.0 was used for the prediction of transmembrane domains (Krogh et al., 2001). The detection of tRNA for protein expression was accomplished using tRNA scan-se 2.0 (Lowe and Chan, 2016). A nucleotide sequence similarity search was conducted in BLASTn using the whole genome sequence of phage vB_SalP_792, and the top 10 hits were retrieved from the BLAST tool. Phages were examined for nucleotide sequence similarity using VIRIDIC (Moraru et al., 2020) using the default settings. A phylogenetic tree was created by comparing the amino acid sequence of RNA polymerase, conserved protein in T7-like phages, with closely related genome hits in BLASTn using MEGA X software.

### Statistical analysis

All the experiments were performed in triplicates and the data were represented as the mean ± standard deviation (SD). Biofilm eradication and Phage lytic activity in the egg were analyzed using *t*-test and the results of the *in vitro* phage kinetics experiment were analyzed using two-way ANOVA. The bacterial reduction in phage-treated groups compared to control (without phage treatment) was considered significant if *p* < 0.05. All the statistical analysis was carried out in GraphPad Prism 8.0 software.

## Results

### Bacteria, phage isolation, and morphology

**Figure 1:**
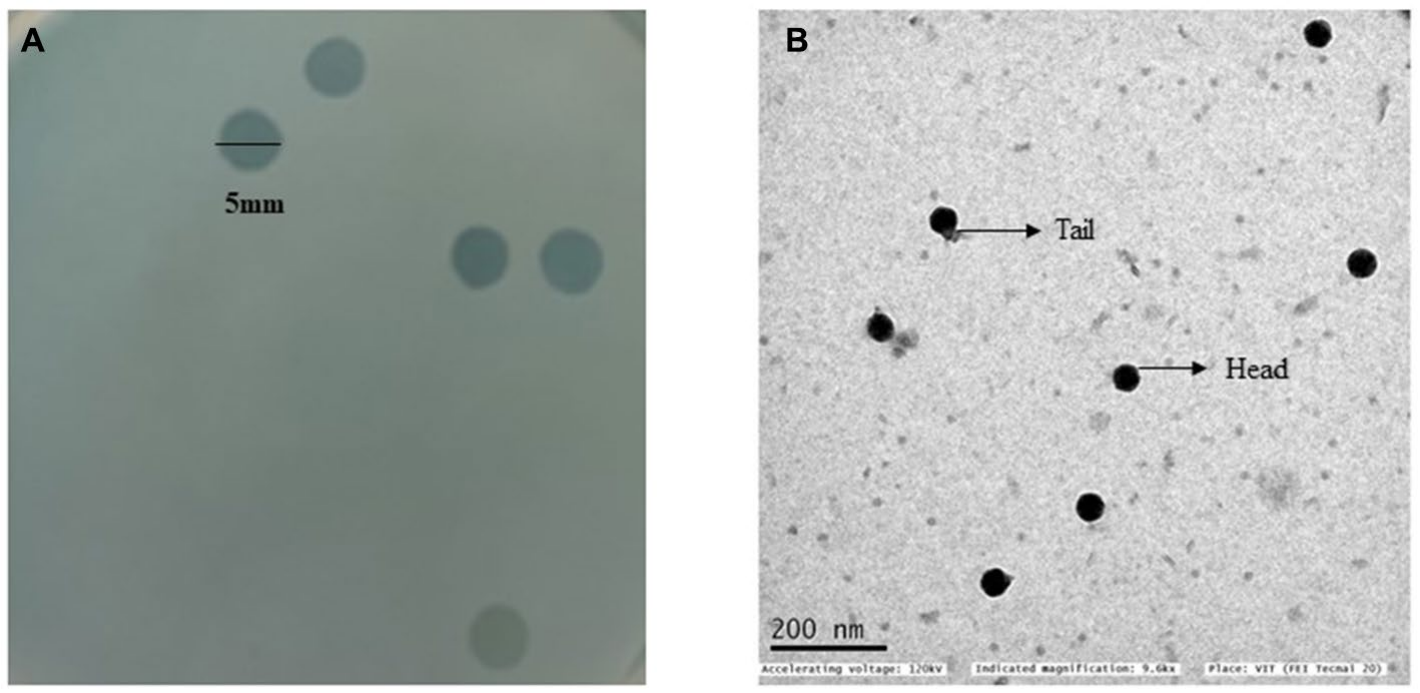
**(A)** A double agar overlay plate with clear plaque morphology. The plaques are round, lytic, and have a diameter of 5 mm. **(B)** A transmission electron microscope (TEM) image of *Salmonella* phage vB_SalP_792 displaying a T7-like morphotype characterized by an icosahedral head and a relatively short tail.

All *S. enterica* isolates in this study showed resistance to cefotaxime (MIC ≥4 μg/mL), and ciprofloxacin (MIC ≥1 μg/mL) (Table 1). A phage that infects the *S. enterica* strain Sal_01 (host) was isolated from domestic sewage water in this study. Phage vB_SalP_792 formed distinct plaques of about 4–5 mm in diameter on the agar overlay plate within 16 h (Figure 1A). The phage vB_SalP_792 was propagated with the host strain Sal_01 for routine studies. TEM investigation revealed that the phage vB_SalP_792 exhibited a T7-like morphology, characterized by an icosahedral head of approximately 45 ± 5.0 nm in diameter and a very short tail (Figure 1B).

**Table 1:** The host range analysis of vB_SalP_792 *Salmonella* phage (Sal-*S. enterica*); “+” indicates Lysis, “-” indicates “no lysis.”

Isolate ID	Cefotaxime MIC value μg/mL	Ciprofloxacin MIC value μg/mL	Host range	EOP
Sal_01 (Host)	8	4	+	3.4 × 10^14^
Sal_02	4	2	+	5 × 10^14^
Sal_03	16	8	+	4.2 × 10^10^
Sal_04	8	4	+	1.05 × 10^11^
Sal_05	4	4	+	9.1 × 10^14^
Sal_06	4	8	+	7.3 × 10^12^
Sal_07	8	2	+	6 × 10^10^
Sal_08	4	8	+	8.5 × 10^8^
Sal_09	8	2	−	
Sal_10	4	1	−	
Sal_11	4	4	−	
Sal_12	4	4	−	
Sal_13	4	2	−	

The efficiency of plating (EOP) of vB_SalP_792 was calculated using a double agar overlay. Cefotaxime MIC values (≥ 4 μg/mL is resistant) and Ciprofloxacin MIC values (≥1 μg/mL is resistant) of 13 *S. enterica* isolates used in the study have been listed.

### Phage host range and EOP

Phage vB_SalP_792 formed plaques against eight out of 13 *S. enterica* isolates and did not form plaques against *E. coli*, *Klebsiella* sp., *A. baumannii*, *P. aeruginosa*, *Citrobacter* sp., *Serratia* sp., *Proteus* sp., *S. aureus*, *Enterococcus* sp., and *Streptococcus* sp. vB_SalP_792 is specialized to infect *Salmonella enterica*. According to the EOP experiments, phage vB_SalP_792 demonstrated high infectivity (EOP >50% infectivity) against all eight isolates listed in Table 1.

### Phage stability

**Figure 2:**
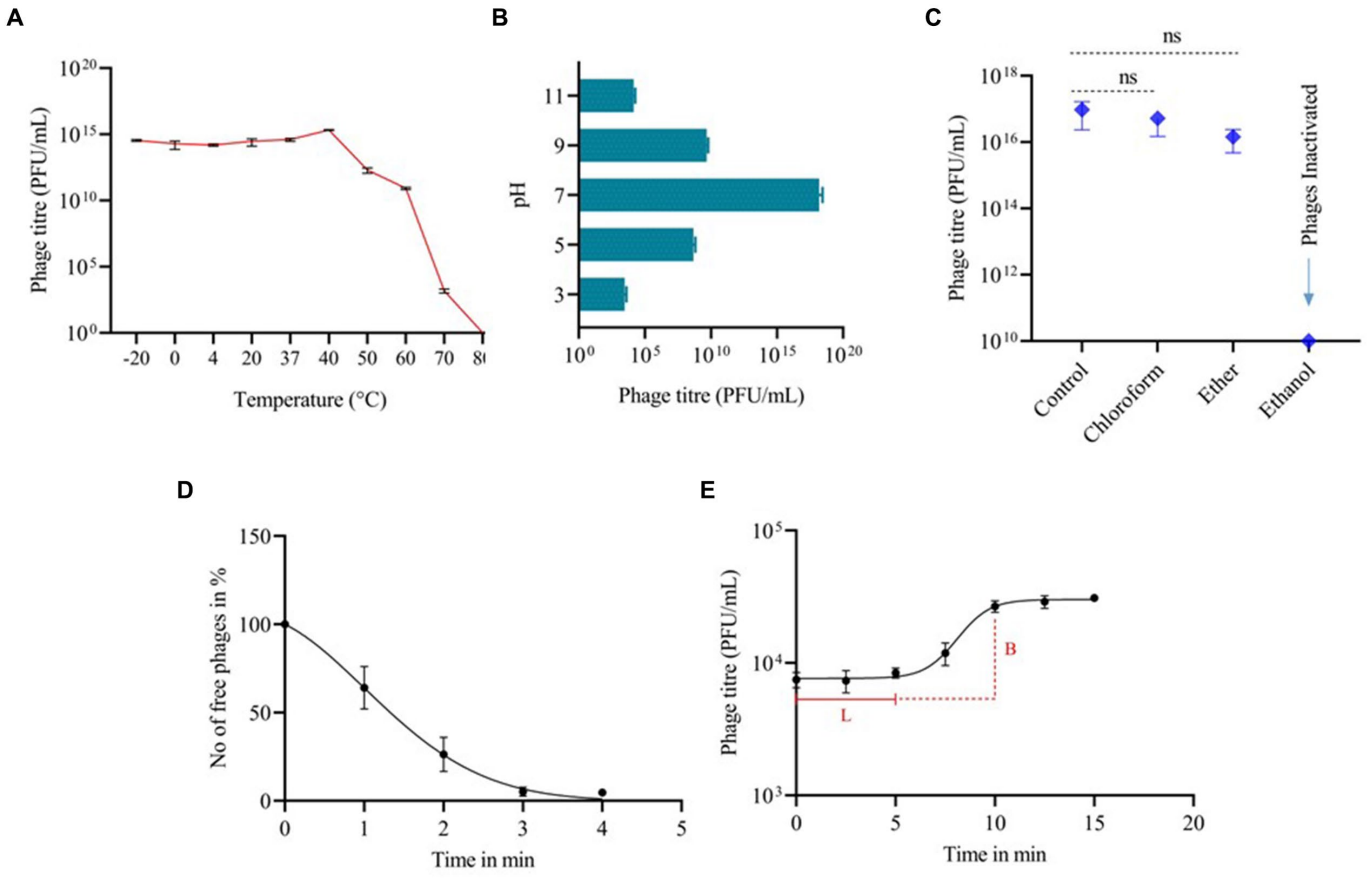
The biological characteristics of *Salmonella* phage vB_SalP_792. **(A)** Thermal stability. **(B)** pH stability. **(C)** Stability in chloroform, ether, and ethanol. **(D)** Phage life cycle focusing on adsorption, and **(E)** one-step growth curve showing a latency period (L) of 5 min and a burst size **(B)** of 50 phages per infected cell. All experiments were conducted in triplicates and the data shown are represented as mean ± SD.

The phage’s stability fluctuated under different pH levels and thermal conditions. Phage vB_SalP_792 exhibited peak activity up to 40°C after 1 h of incubation, with no significant difference (*p* < 0.05) observed in phage stability between −20 and 40°C temperatures (Figure 2A). The phage activity decreased at 70°C and was inactivated at 80°C. After 1 h of incubation, the phage was discovered to be thermotolerant at 60°C (Figure 2A). In terms of pH stability, the peak activity was observed at pH 7 following a 1-h incubation at 37°C. The phage exhibited decreased stability at pH 3 (acidic) and pH 11 (alkaline) as shown in Figure 2B. In solvent stability, there was no significant decrease in the titer of the phage incubated with chloroform and ether compared to the control, whereas phage vB_SalP_792 was inactivated in ethanol (Figure 2C).

### One-step growth curve

Phage vB_SalP_792 achieved a 95% adsorption rate to its bacterial host within 3 min, which is the peak adsorption rate (Figure 2D). The one-step growth curve revealed that the phage had a short latent phase of 5–7 min, followed by a rising period of up to 5 min. The progeny production rate (burst size) was approximately 50 phage particles per infected cell (Figure 2E). The phage life cycle was found to be 10 min under *in vitro* conditions.

### Genome analysis

The genome of *Salmonella* phage vB_SalP_792 is dsDNA, comprising 37,218 bp in length, with a G + C composition of 51% (Grant and Stothard, 2008; Supplementary Figure S1). 3,862,522 high-quality reads of 150 bp each (in paired-end format) were used for constructing the phage genome. There are 42 Open Reading Frames (ORFs) found in the genome, with 25 having known functions and 17 being hypothetical. The PhageTerm analysis revealed that vB_SalP_792 utilizes T7 packaging and possesses a direct terminal repeat (DTR) of 192 bp. The phage is categorized under the *Autographiviridae* family and is part of the unclassified *Kayfunavirus* genus. The genes holin, spanin, and endolysin, which are connected to host lysis, are located on the positive strand. The genome shared an average amino acid identity of 95.8% with *Salmonella* phage vB Seyj3-1 (MW416012). vB_SalP_792 lifestyle is virulent as foreseen by PhaBOX. No resistance genes or tRNAs were found. vB_SalP_792 lacks lysogenic genes, making it obligately lytic, safe, and a promising candidate for phage therapy.

The nearest BLASTn matches shared the same classification as unclassified *Kayfunavirus* and were selected for the intergenomic study using VIRIDIC (Supplementary Figure S2A). The vB_SalP_792 is 98.1% identical to ST38. The conserved protein RNA polymerase amino acid sequence was examined using the MEGA X program (Supplementary Figure S2B). Strains such as ST64 (OQ860964.1), ST59 (OQ860966.1), and ST63 (OQ860965.1) are closely linked with no genetic distance between them, suggesting they are genetically identical for RNA polymerase and phage replication. vB_SalP_792 shows a notable genetic distance from other strains, with a branch length of 0.012, suggesting genetic divergence.

### *In vitro* assays

**Figure 3:**
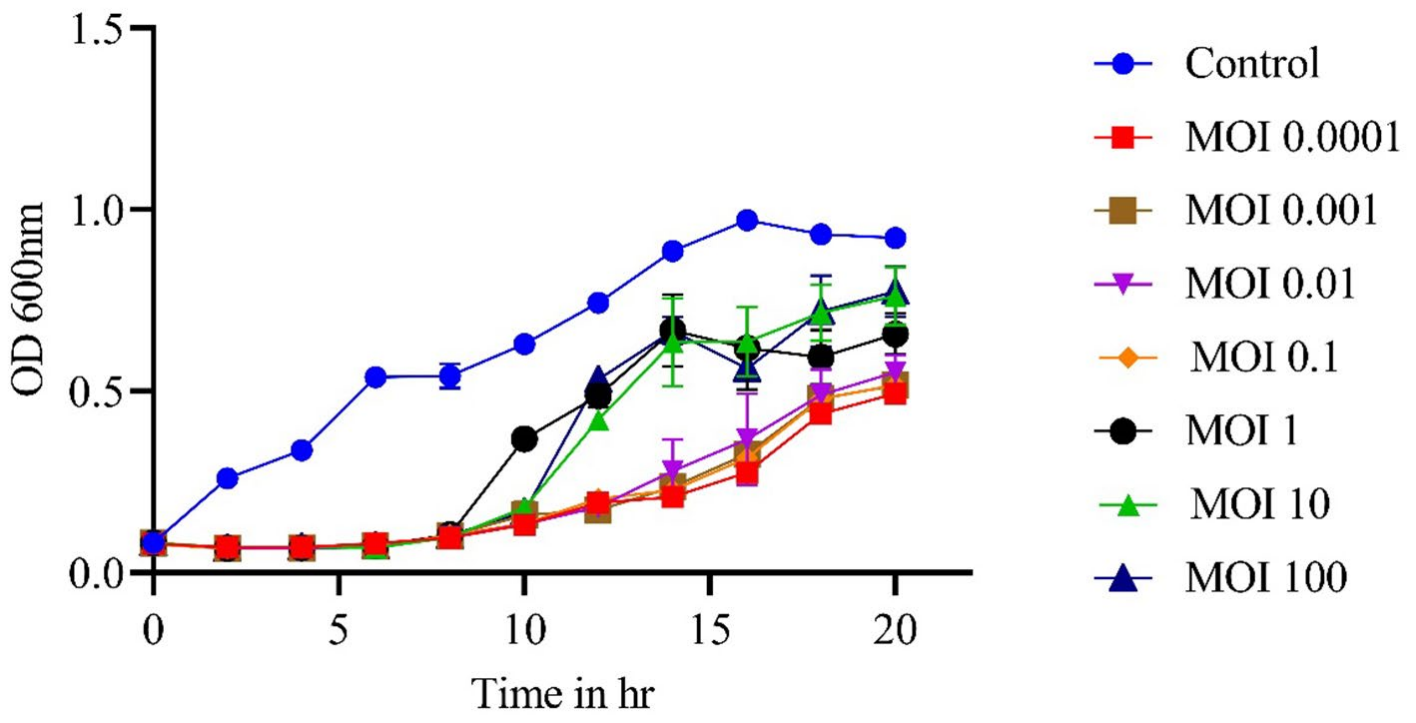
*In vitro* phage kinetics experiment used to assess bacterial inhibition. Phage vB_SalP_792 demonstrated strong lytic activity against planktonic cells at varying MOIs ranging from 0.0001 to 100. All experiments were performed in triplicates and the data shown are represented as mean ± SD.

The phage-killing experiment showed a significant reduction in bacterial growth in groups treated with phages compared to the control group (*p* < 0.0001), indicating the effectiveness of the phage vB_SalP_792 *in vitro*. Until the 6th hour, all the different MOIs ranging from 0.0001 to 100 exhibited comparable levels of growth inhibition. Adsorption levels were lower after 10 h with MOIs 1, 10, and 100 compared to MOIs of 0.1, 0.01, 0.001, and 0.0001 (Figure 3). Bacterial growth decreased at 10 and 15 h when the MOIs were 0.1, 0.01, 0.001, and 0.0001. The phage had a very short life cycle and effectively inhibited bacteria for up to 15 h. However, resistant variants appeared after 20 h (no further studies were performed).

### Inhibition of bacterial growth in chicken egg white

**Figure 4:**
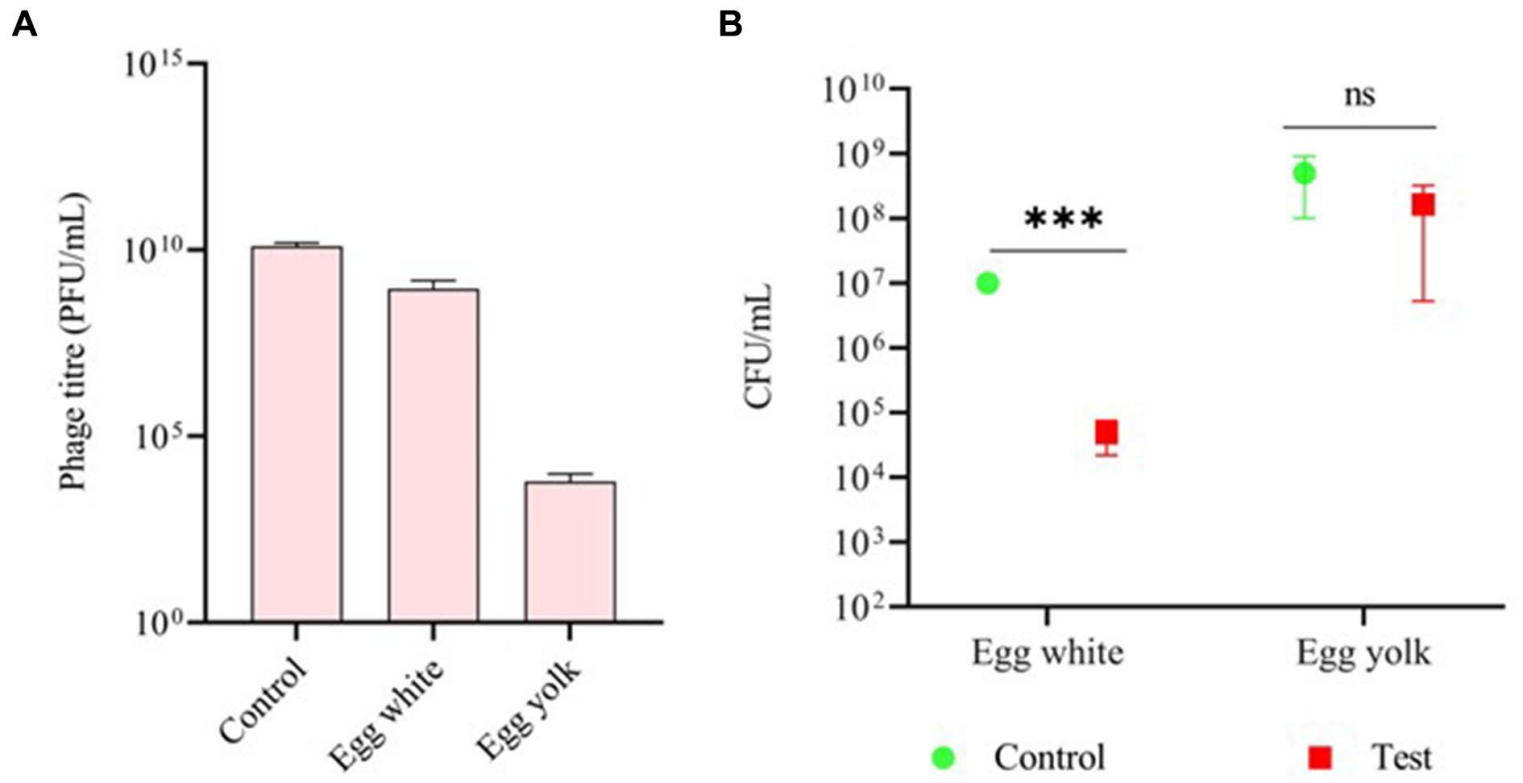
Assessing the efficacy of phage vB_SalP_792 using a chicken egg. **(A)** Investigating the stability of phage vB_SalP_792 in egg white and egg yolk, as well as **(B)** its lytic activity in egg white and yolk. All experiments were performed in triplicates and the data shown are represented as mean ± SD. *p* ≤0.05 compared to the control. Control groups solely include bacteria found in egg whites and yolk. (***p ≤0.001).

The stability of phage vB_SalP_792 was assessed in both egg white and egg yolk. The phage titer in egg white (10^8^) was very steady for 1 h compared to the control (10^10^). The titer in the egg yolk was decreased by twofold (10^4^) (Figure 4A). The phage had a significant inhibitory impact on egg white (*p* ≤ 0.00001), resulting in a decrease in bacterial load. The egg yolk did not demonstrate significant bacterial reduction (*p* = 0.4), indicating lower phage activity, which aligns with the stability test results (Figure 4B).

### Antibiofilm activity of vB_SalP_792 phage

**Figure 5:**
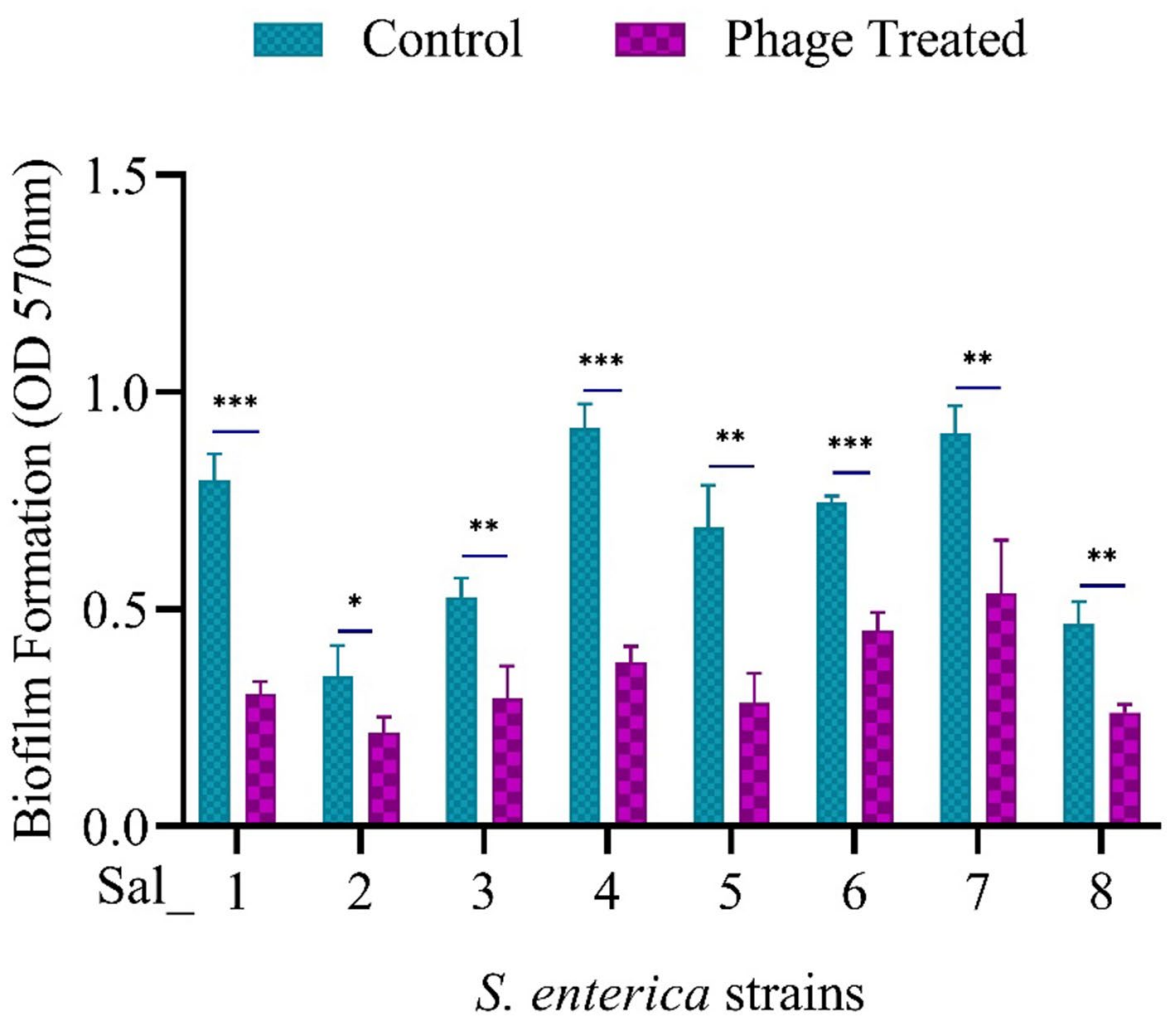
Population of *Salmonella enterica* biofilm before and after phage treatment. 24-h-old biofilm cells were exposed to *Salmonella* phage vB_SalP_792 and incubated at 37°C for 24 h. The results were then measured at OD 570 nm using a microtiter plate reader (CV assay). All experiments were performed in triplicates and the data shown are represented as mean ± SD. p <0.05 compared to the control. (***p ≤ 0.001, **p ≤ 0.002).

Prior to phage treatment, a biofilm of *S. enterica* was cultured and allowed to grow individually at 37°C for 24 h. All eight isolates were found to develop biofilms, as shown in the control in Figure 5. The effectiveness of phage vB_SalP_792 in eradicating biofilm was assessed by analyzing the alterations in the biofilm population using a CV assay. After treating the biofilms with phage (100 μL at 1 × 10^9^ PFU/mL), a notable decrease in biofilms was observed within 24 h compared to the control group (Figure 5). vB_SalP_792 demonstrated notable anti-biofilm activity, as evidenced by the substantial decrease in bacterial load (*p* < 0.05).

## Discussion

Phages are the most prevalent life forms on Earth. Bacteria in nature are predominantly infected by phages, making it possible to isolate phages based on the bacterial habitat. Phage-bacterial interaction occurs randomly, although phages exhibit high specificity toward their host. Host specificity is one of the characteristics of phages, making them valuable in several fields such as the food industry, medicine, and agriculture. Utilizing bacteriophages for biocontrol of food products is a newly investigated area. *Salmonella* phage products are currently utilized as effective food biocontrol agents in the market. There is a rising interest in using them therapeutically because of the escalating antibiotic resistance. Studies highlighting resistance to fluoroquinolones, third-generation cephalosporin (Hooper, 2001; Raveendran et al., 2008) and last-resort treatments such as colistin (Lima et al., 2019; Fortini et al., 2022) emphasize the need to develop a treatment plan to prevent *Salmonella* from becoming ESKAPE pathogens (*Enterococcus faecium, Staphylococcus aureus, Klebsiella pneumoniae, Acinetobacter baumannii, Pseudomonas aeruginosa*, *and Enterobacter species*) which causes major multidrug-resistant nosocomial infections (WHO, 2021).

This study investigated the biological features of a *Salmonella* phage vB_SalP_792 that has potential applications in the food sector and clinical settings. Multi-drug resistant *Salmonella* strains are being studied to evaluate the effectiveness of phages. Phage vB_SalP_792, isolated from sewage water samples, belonging to T7-like phages recognized by microscopic analysis, demonstrated a wide host range by infecting eight out of 13 clinical *S. enterica* strains that are resistant to cefotaxime and ciprofloxacin. *Salmonella* phages previously identified were found to have a limited range of hosts, however T7-like phages with a wider host range have also been reported (Zhang et al., 2021; Cao et al., 2022; Mondal et al., 2022). Phage vB_SalP_792 was tested for their infectivity against other gram-negative and gram-positive clinical pathogens such as *E. coli*, *K. pneumoniae*, *A. baumannii*, *P. aeruginosa*, *Serratia*, *Citrobacter*, *Proteus*, *S. aureus*, *Streptococcus*, and *Enterococcus* and was recognized as *Salmonella* specific/Genus specific. A broader host range in the phages within the genus/species helps in lysing various serotypes and sequence types of the same species without affecting the normal gut microbiota which makes them advantageous over antibiotics since most of the first-line antibiotics used are broad-spectrum (Ross et al., 2016). High efficiency of plaquing against all the host range positive *Salmonella* isolates, demonstrates vB_SalP_792 phage having high infectivity against its targeted genus.

The morphological analysis uncovered that the vB_SalP_792 consists of a head/capsid and a short tail. The phage capsid is made up of proteins that protect the genetic material and a tail that serves to attach the phage to the bacterial cell surface receptors. Genomic analysis revealed that the vB_SalP_792 phage belongs to the class of *Caudoviricetes*, also known as tailed phages, the class that represents the highest percentage of the phage sequences submitted in the NCBI database (Guttman, 2013; Moraru et al., 2022). The isolated T7-like phage from the *Autographiviridae* family has an exceptionally short life cycle of 10 min and a rapid adsorption rate of 95% within 3 min. The latency of *Salmonella* phages PSDA-2 (Sun et al., 2022), STGO-35-1 (Rivera et al., 2022), and vB_SalP_TR2 (Zhang et al., 2021) previously documented had 10, 30, and 15 min, respectively. Phage vB_SalP_792 had a shorter adsorption and latent period compared to other *Salmonella* phages (Zhang et al., 2021; Rivera et al., 2022; Sun et al., 2022) which makes it a potential candidate for phage therapy.

The stability of the phages is important for optimizing the route of administration and storage conditions. The tolerance to extreme acidic pH conditions indicates that vB_SalP_792 phage can be administered orally. The phage was highly stable at various temperatures from −20 to 40°C, this allows the easy transportation and storage of vB_SalP_792 phage for a long period, without affecting its titer. The stability results correlate with other *Salmonella* phages reported previously (Shang et al., 2021; Zhang et al., 2021). There was a decrease in titer from 50°C, but the phage exhibited strong heat resistance up to 70°C which is similar to other *Salmonella* phage LPST153 (Islam et al., 2020). Ongoing research is being conducted to verify the ability of phage vB_SalP_792 to infect at high temperatures of up to 70°C. The phage titer was not affected by the exposure to solvents such as chloroform and ether, whereas completely inactivated in ethanol. The solvent stability of phages has to be examined since organic solvents have an impact on the integrity of proteins, and solvents are majorly used during the preparation of phage formulation (Srot et al., 2016).

One novel method to address foodborne infections is utilizing lytic phages as a biocontrol agent. ListShield™, developed by Intralytix Inc. United States, is the initial phage-based solution authorized for managing *L. monocytogenes* in meat and poultry (Endersen et al., 2014). Additional products include Ecoshield™ for combating *E. coli* 0157: H7 in food processing facilities and SalmoFresh™ for addressing *Salmonella* in red meat and poultry. Various studies are being conducted to develop phage-based products for biocontrol purposes, targeting pathogens such as *E. coli* 0157:H7 in meat (O'Flynn et al., 2004), lettuce, beef (Sharma et al., 2009)*, Campylobacter* in chicken skin (Atterbury et al., 2003), raw chicken meat (Orquera et al., 2012), and cooked beef (Bigwood et al., 2009), *Listeria monocytogenes* in fruits and cheese (Leverentz et al., 2001), *Salmonella* in cheese (Modi et al., 2001), fruit (Leverentz et al., 2003), chicken (Whichard et al., 2003), cooked beef, chocolate milk, hot dogs, seafood (Guenther et al., 2012), and fresh eggs (Połaska and Sokołowska, 2019). These types of applications of bacteriophages as biocontrol agents replace the use of antimicrobials or other preservatives added to increase the shelf-life of a food product, which usually affects the quality of the food and is unsafe, therefore bacteriophage use in the food industry is considered as green technology as it is naturally derived and does not have any harmful effects on consumption, this also reduces economic loss caused by spoilage and food contamination (Gildea et al., 2022).

In this study, the phage vB_SalP_792 was examined for its stability and lytic activity in eggs to evaluate its utilization as a biocontrol agent in food applications. After 1 h of phage incubation in egg white and egg yolk separately, the phage titer was reduced compared to the control but the titer remained stable in egg white than in egg yolk, this can be because of the formation of phage-protein or phage-fat complexes or the presence of anti-phage proteins in egg yolk (He et al., 2024). The stability of phages in food matrices is complex as it is influenced by a variety of components in the food (García-Anaya et al., 2020), the formation of these phage complexes has already been reported in a study conducted in milk (Das and Marshall, 1967). The presence of a high quantity of proteins and lipids in the yolk might be a reason for the lower phage stability compared to egg whites which contain 90% water.

The phage vB_SalP_792 was able to reduce the bacterial load more effectively in egg whites than in egg yolk, possibly because the phage activity was hindered by the denser composition of egg yolk as compared to the less dense egg white (Mehta et al., 2021). A study conducted by Jiangning demonstrates the difficulty in treating bacterial contamination in egg yolk compared to egg whites, with egg yolk needing for longer treatment duration, higher MOIs up to 1,000, and lesser reduction in bacterial load. The difference in the phage lytic activity was explained as the nature of viscosity between egg white and egg yolk, where in egg white the phage motility was not affected and the phage adsorption to bacterial receptors was easier. The nutrients in the egg yolk helped in bacterial multiplication but disabled phage motility (He et al., 2024). Earlier studies have reported the efficiency of *Salmonella* phage Pu20 showing lytic activity against drug-resistant *Salmonella* in liquid eggs (Zhang et al., 2021). A similar study found that treating a fresh egg with a cocktail of *Salmonella* phages resulted in a slight decrease in the bacterial load (Spricigo et al., 2013). In a study conducted by Leverentz et al., phages applied on watermelon slices maintained their titer and showed effective lytic activity, compared with phages applied on apple slices where phages became invisible after 48 h. This difference in phage stability and activity is because of the slightly acidic nature of apples (pH 4.2) when compared with melon (pH 5.8) (Leverentz et al., 2001). In many food preserving procedures, the phage needs to remain stable at various pH conditions of that of food, the phage vB_SalP_792 remained stable at neutral pH and remained effective in acidic (pH 3) and alkaline conditions (pH 11) for up to an hour. This makes it a promising candidate for usage in several food processing applications (Li et al., 2020; Shang et al., 2021).

The genome of vB_SalP_792, which is 37,281 bp long, is similar to the formerly reported *S. enterica* podoviridae SE20 phage having 40,000 bp (Dallal et al., 2019) and much shorter than other podoviridae *Salmonella* phages such as Pu20 having 59,435 bp (Zhang et al., 2021), vB_SalP_TR2 having 71,453 bp (Shang et al., 2021), and pSal-SNUABM-01 having 80,500 bp (Kwon et al., 2022). The vB_SalP_792 phage shares a high similarity (up to 95%) with known phage genomes in the database such as ST38 (OQ860974.1), ST63 (OQ860965.1), ST66 (OQ860962.1), ST64 (OQ860964.1), ST29 (OQ860978.1), ST59 (OQ860966.1), ST16 (OQ860982.1), and ST17 (OQ860981.1), all of which were isolated and reported from Nepal. All these closely related *Salmonella* phages are identified from the Indian subcontinent. A phylogenetic tree constructed for the terminase large-subunit shows significant genetic diversity in the terminase large-subunit of vB_SalP_792 compared to other phage terminases. Moreover, the lack of tRNA suggests that vB_SalP_792 depends on the host’s machinery for protein transcription. This trait could potentially enhance the phage’s specificity and efficacy by preventing replication in the absence of a suitable host. The genome nature of vB_SalP_792 phage is comparable to most of the lytic phages reported earlier (Cong et al., 2021; Shang et al., 2021; Zhang et al., 2021).

The genome investigation of vB_SalP_792 phage revealed the presence of structural genes such as head-to-tail connector protein (WQZ00366.1), capsid and scaffold protein (WQZ00367.1), major capsid protein (WQZ00368.1), tail fiber protein (WQZ00370.1), and non-contractile tail tubular protein (WQZ00372.1). It also consists of genes responsible for DNA packaging and replication including terminase large subunit (WQZ00339.1) and DNA-directed DNA polymerase (WQZ00357.1). The presence of holin and endolysin genes shows that vB_SalP_792 uses a Holin-Endolysin cassette for lysis (Cong et al., 2021). The vB_SalP_792 genome’s lack of genes linked to antibiotic resistance, virulence, toxicity, or lysogeny makes it an obligately lytic phage, a promising candidate for clinical applications. Because, phages carrying any of the aforementioned genes if used for treatment can enhance the severity of infection by adding virulence and toxicity to the existing bacterial infection, thereby complicating treatment. Further study is needed to comprehensively characterize the genomic features of vB_SalP_792 and confirm its therapeutic potential for phage therapy applications. The multiplicity of infection is the ratio of phage to bacteria, where 0.0001 is the lowest concentration of phage and MOI 100 is the highest. The phage-killing assay demonstrated considerable inhibition of bacterial growth at low MOI values of 0.0001, 0.001, 0.01, and 1. At MOIs <1, even though the phage concentration is low, based on the phenomenon of auto-dosing, bacterial lysis happens. Proficient phage lytic activity at lower MOIs <1 has been reported in past studies (Raj, 2022; Abdelsattar et al., 2023). At higher MOIs >1 though there will be an immediate reduction in the bacterial density due to a higher ratio of phages than bacteria, if the host develops phage resistance, a scarce population of phage-resistant mutants thrives, which results in the regrowth of bacteria (Egido et al., 2022). Though there are studies reporting phage lytic activity in all MOIs without bacterial regrowth/phage resistance for even 12 h (Islam et al., 2020), in our study bacterial growth increased significantly at increasing MOIs of 1, 10, and 100 after 6 h, possibly due to the emergence of phage resistance preventing phage attachment. Previous studies have shown that bacteria regenerate after a decrease in phage at higher MOIs due to phage-resistant mutants (Zulk et al., 2022). Thus, administering several doses of smaller MOIs of phage, usage of phage-antibiotic synergy, or phage cocktails can be used to prevent the development of phage resistance (Abdelsattar et al., 2023).

Biofilm formation is one of the major resistance mechanisms in bacteria, it forms a structural barrier for the entry of antibiotics, enclosing themselves within the extracellular polymeric substances. Biofilm-forming bacterial inhabitation in food manufacturing materials and equipment affects food quality and safety leading to contamination (Abebe, 2020). Food-borne pathogens like *E. coli* and *Salmonella* lead to cross-contamination from food processing environments to food products affecting human health (Lee et al., 2021). The phage vB_SalP_792 effectively decreased preformed biofilm load in all studied isolates, with the highest reduction of 1.79-fold observed in the Sal_04 strain. This aligns with the previous research on the antibiofilm activity of phages (Chaudhary et al., 2022; Hosny et al., 2023). Phage produce depolymerases and lysins that aid in breaking down exopolysaccharides, a key component of biofilms (Chegini et al., 2020). Previous investigations have indicated that phages can eliminate biofilms, especially with depolymerases (Hosny et al., 2023). The tail fiber protein (ORF42) is associated with the anti-biofilm activity of vB_SalP_792. The ORF42 (WQZ00377.1) was predicted to have an N-terminal tail fiber protein and a C-terminal carbohydrate esterase (CE) domain from amino acids 219 to 498, which plays a role in degrading EPS. Previously reported phages like LPSTLL, LPST94, and LPST153 shown significant reduction in *Salmonella* biofilm formed in 96-well plates and stainless-steel surfaces (Islam et al., 2019). Though the static biofilm does not exactly mimic the dynamic environment where biofilm usually forms in the body, investigating the biofilm formations of clinical pathogens and antibiofilm properties of phages *in vitro* provides basic insights to understand the host-phage interaction and also to optimize the phage dosage during administration, these findings will help in translating benchwork to *in vivo* or in flow studies.

*In vitro* laboratory-based characterization and genomic analysis of bacteriophages is as important as *in vivo* or pre-clinical studies, to eliminate lysogenic and inefficient phages, thereby saving resources and time. The phage vB_SalP_792 has proved itself to be efficient in eliminating *Salmonella* bacterial load in *in vitro* conditions even at lower MOIs like 0.0001 as well as in food sources. During administration, the optimum concentration of bacteriophages has to be maintained and reach the infected area enduring various physiological conditions to eliminate the bacteria from the body, since the decrease in the phage titer may lead to sub-therapeutic doses (Uyttebroek et al., 2023). Stability is a prerequisite during formulations to manufacture phage products. The vB_SalP_792 phage’s ability to withstand various stress conditions like temperature, pH, and solvents, shows its suitability for *in vivo* testing, and for practical implementation. However, the phage vB_SalP_792 can be used as an effective food biocontrol agent and also in treating patients, it is effective against only *Salmonella* infections. In the case of polymicrobial infections, a cocktail of phages can be adopted along with vB_SalP_792 to acquire a broader host range against pathogens.

## Conclusion

The emergence of antibiotic resistance, especially in food-borne pathogens is threatening and increases the risk of transmission. Although, the usage of phage-based products in food industries to avoid contamination is in practice, treating multi-drug resistant infections with obligately lytic phages is an emerging trend. This study highlights the efficiency of Vb_SalP_792 phage in eliminating clinical strains of *S. enterica* isolates in *in vitro* conditions, with potent anti-biofilm activity, it also reduces the bacterial load in egg whites. Further, *in vivo* studies have to be performed to understand the pharmacokinetics and pharmacodynamics of the phage.

## Data availability statement

The assembled sequence is submitted in the NCBI database with an accession number OR978128. The raw reads are also submitted in the NCBI-SRA database with an accession number SAMN38857082.

## Author contributions

RJ: Writing – original draft, Writing – review & editing, Formal analysis, Methodology. AL: Methodology, Resources, Validation, Writing – review & editing. AN: Methodology, Writing – review & editing. YB: Data curation, Formal analysis, Writing – review & editing. BV: Conceptualization, Data curation, Project administration, Resources, Supervision, Writing – review & editing. PM: Conceptualization, Data curation, Project administration, Resources, Supervision, Writing – review & editing. RN: Conceptualization, Data curation, Project administration, Resources, Supervision, Writing – review & editing.
