# Supplementary material for: Characterization of Salmonella phage of the genus Kayfunavirus isolated from sewage infecting clinical strains of Salmonella enterica
Source: Front Microbiol. 2024 Jun 3;15:1391777. doi: 10.3389/fmicb.2024.1391777 (PMC11180730; doi:10.3389/fmicb.2024.1391777)

Supplementary Material

Characterization of *Salmonella* phage of the genus *Kayfunavirus* isolated from sewage infecting clinical strains of *Salmonella enterica*

*Ramya Juliet[1], Archana Loganathan[1], Ayyanraj Neeravi[2], Yamuna Devi Bakthavatchalam[2], Balaji Veeraraghavan[2], Prasanth Manohar[3,4], Ramesh Nachimuthu[1]**

[1]School of Biosciences and Technology, Vellore Institute of Technology, Vellore, 632014, India

[2]Department of Clinical Microbiology, Christian Medical College, Vellore, 632004, India

[3]Department of Biochemistry and Biophysics, Texas A&M AgriLife Research, Texas A&M University, College Station 77843-2128, Texas, USA

[4]Center for Phage Technology, Texas A&M AgriLife Research, Texas A&M University, College Station 77843-2128, Texas, USA

**Correspondence:*

Dr. Ramesh Nachimuthu: ramesh.n@vit.ac.in

Supplementary Figure 1. The genome map of the phage vB_SalP_792 was constructed using the CGView server.

Supplementary Figure 2. (A) Illustrates the classification of the nucleotide sequences of phage vB_SalP_792 with respect to 10 closely related phages using VIRIDIC. The heatmap illustrates the similarity scores between the phages' genomes. The matrix displays percentage values representing the similarity between pairs of bacteriophage sequences in each cell. **(B)** Phylogenetic analysis was conducted on the RNA polymerase protein of vB_SalP_792 and its closely related phages using BLAST.

Supplementary Figure 1

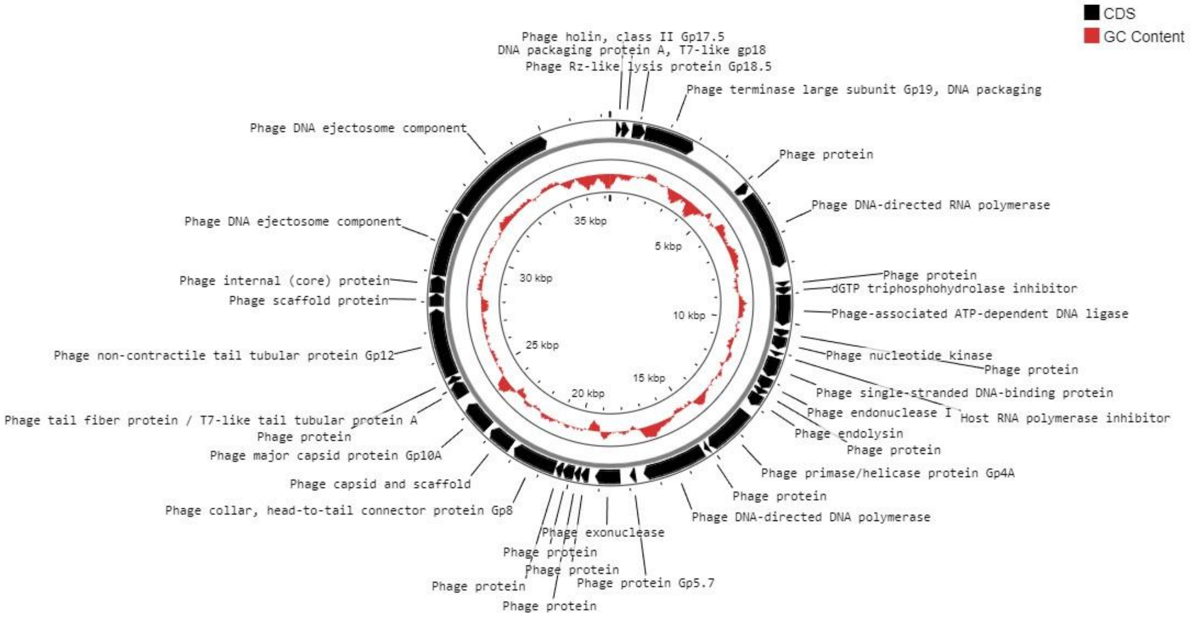

Supplementary Figure 2

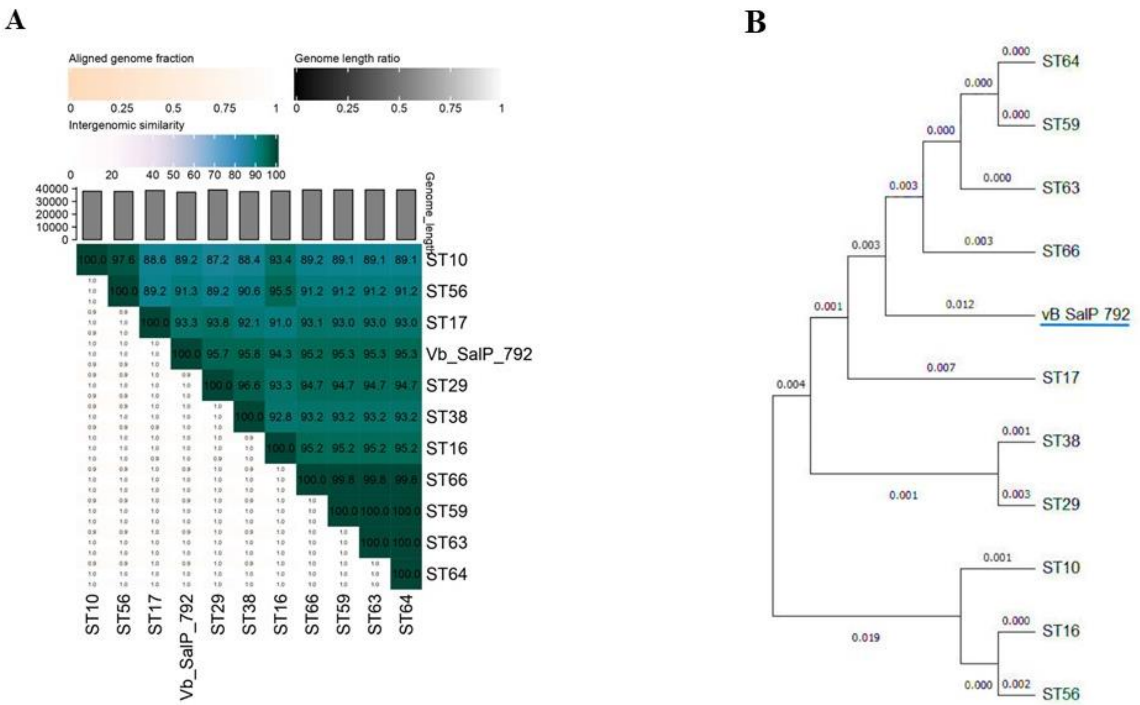

Supplement: Supplementary file 1 [file Data_Sheet_1.PDF]
